# Stochastic Mesocortical Dynamics and Robustness of Working Memory during Delay-Period

**DOI:** 10.1371/journal.pone.0144378

**Published:** 2015-12-04

**Authors:** Melissa Reneaux, Rahul Gupta, Karmeshu

**Affiliations:** 1 School of Computational and Integrative Sciences, Jawaharlal Nehru University, New Delhi, India; 2 School of Computer and Systems Sciences, Jawaharlal Nehru University, New Delhi, India; SUNY Downstate MC, UNITED STATES

## Abstract

The role of prefronto-mesoprefrontal system in the dopaminergic modulation of working memory during delayed response tasks is well-known. Recently, a dynamical model of the closed-loop mesocortical circuit has been proposed which employs a deterministic framework to elucidate the system’s behavior in a qualitative manner. Under natural conditions, noise emanating from various sources affects the circuit’s functioning to a great extent. Accordingly in the present study, we reformulate the model into a stochastic framework and investigate its steady state properties in the presence of constant background noise during delay-period. From the steady state distribution, global potential landscape and signal-to-noise ratio are obtained which help in defining robustness of the circuit dynamics. This provides insight into the robustness of working memory during delay-period against its disruption due to background noise. The findings reveal that the global profile of circuit’s robustness is predominantly governed by the level of D1 receptor activity and high D1 receptor stimulation favors the working memory-associated sustained-firing state over the spontaneous-activity state of the system. Moreover, the circuit’s robustness is further fine-tuned by the levels of excitatory and inhibitory activities in a way such that the robustness of sustained-firing state exhibits an inverted-U shaped profile with respect to D1 receptor stimulation. It is predicted that the most robust working memory is formed possibly at a subtle ratio of the excitatory and inhibitory activities achieved at a critical level of D1 receptor stimulation. The study also paves a way to understand various cognitive deficits observed in old-age, acute stress and schizophrenia and suggests possible mechanistic routes to the working memory impairments based on the circuit’s robustness profile.

## Introduction

Working memory is extremely crucial for the performance of various cognitive tasks [1–3]. Though its functioning involves the activation of multiple regions of the cortex, the dorsolateral prefrontal cortex (DLPFC) plays a pivotal role in the maintenance and manipulation of working memory [4, 5]. Neurophysiological studies have shown that neurotransmitters, such as dopamine, exert a profound effect on the working memory formation [6, 7]. Studies involving delayed-response tasks have demonstrated the role of dopamine in the modulation of spatial working memory in monkeys through the activation of D1 receptors [8–10]. Ageing [11], stress [12] and various neuropsychiatric disorders, such as schizophrenia [13, 14], are commonly characterized with impaired working memory and have been found to be intricately connected with the abnormal dopamine content of the prefrontal cortex [15–17]. Many such findings have made working memory and its dopaminergic modulation a subject of great interest. Significant efforts, involving experimental [18–22] and computational approaches [23–28], have provided an insight into the mechanism of working memory formation and its manipulation. Based on the computational studies of working memory involved in spatial tasks with multiple targets [29, 30], Tanaka proposes the operational control hypothesis of dopamine to elucidate the role of dopamine in the processing of working memory.

The operational control hypothesis postulates that the fundamental cognitive operations are controlled by dopamine through its effect on the level of D1 receptor activation and emphasizes the role of mid-brain dopaminergic neurons in the regulation of dopamine content in the DLPFC through the open- and closed-loop controls. The loop represents the reciprocal functional interaction embedded in the mesocortical circuit design. The open-loop control involves stimulation of dopamine nuclei from external inputs other than the mesocortical circuit which may lead to an increase in the dopamine content of the PFC and affects the updating of working memory. On the contrary, the closed-loop control is predominantly engaged in the maintenance of working memory. Here, activity of the DLPFC involved in the working memory representation itself regulates release of dopamine from the mid-brain region through a feed-back control in the closed-loop mesocortical circuitry. To illustrate how the closed-loop control works, a mathematical model of the circuit has been proposed earlier [28] which incorporates essential interactions among the circuit components into a dynamical framework. This framework demonstrates the formation of circuit’s operating points on the typically observed inverted U-shaped profile of working memory modulation by dopamine.

The model involves two crucial parameters: dopamine releasabilty in the DLPFC via the dopaminergic afferents and the efficacy of the corticomesencephalic glutamatergic transmission (excitatory projections from DLPFC to midbrain), and demonstrates that alterations in these parameters may lead to impaired working memory. Also, it suggests a plausible mechanism for the inconsistent DLPFC activity observed in schizophrenia. Here, it should be mentioned that the model furnishes a qualitative picture of the process based on minimal number of essential interactions required to effectively capture the essence of the circuit’s functioning. The goal of the model is not to achieve quantitative resemblance to experimentally obtained data in real biological scenario. Although there are numerous highly sophisticated and detailed models [23, 25, 26] presently available which employ neural network approaches to address this process, this model owing to its simplicity as well as effectiveness provides a useful framework for the study of the closed-loop mesocortical circuit.

However, the deterministic description of the circuit dynamics ignores the role of noise and its impact in bringing out new features of the dynamical system. It is a well-known fact that there are inherent random fluctuations present in the variables of interest in the brain [31, 32] which can significantly affect the properties of the circuit [33]. The constantly evolving and changing properties of the environment collectively serve as one of the potential sources of noise in the mesocortical circuit. Therefore, for a more realistic description of the dynamical system, it is imperative to formulate the model of the circuit dynamics in the stochastic framework. This analysis will provide insights into both the evolutionary as well as the steady state properties of the underlying variables. In this regard, our interest is to investigate the steady state properties of the mesocortical circuit. For this, global potential energy landscape of the circuit dynamics is constructed to address the issue of the stability of working memory formed during the delay period in the presence of noise.

The present study leads to the emergence of many interesting features associated with the closed-loop mesocortical circuit that otherwise remained unseen in the deterministic framework. The study demonstrate a fundamental role of D1 receptor activity in the robustness of working memory during the delay period. Also, it points towards the possibility of a fine-tuning of the circuits robustness by the relative levels of excitatory and inhibitory activities underlying the establishment of sustained firing associated with the working memory representation. Eventually, it provides a platform to investigate how noise-perturbed dynamics of the mesocortical circuit under abnormal dopamine conditions engender the various cognitive deficits observed in old-age, acute stress and schizophrenia. Therefore, the stochastic formulation has led to the enhancement of the applicability of the previously proposed deterministic model.

## Materials and Methods

### The Model

**Fig 1:**
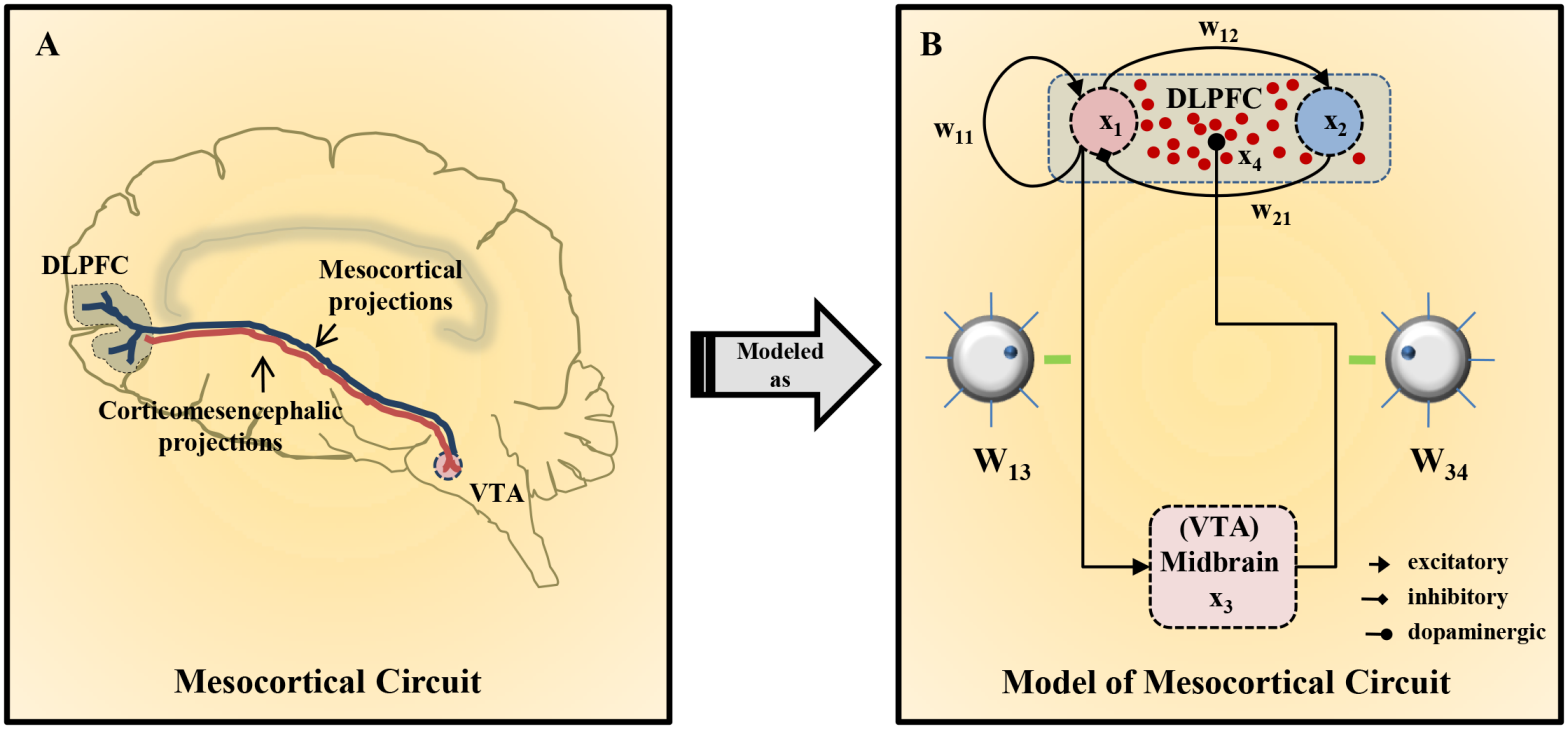
Schematic depiction of the Mesocortical circuit. (A) The interactions of the DLPFC and VTA in the central nervous system. DLPFC (grey region) innervates VTA nuclei (pink) located in the midbrain through corticomesencephalic glutamatergic projections (red). Reciprocally, VTA sends dopaminergic mesocortical projections (blue) to the DLPFC. (B) Detailed connection profile of various functioning modules in the mesocortical circuit. In the DLPFC, the group of pyramidal neurons, *x*_1_ (red), excites itself through recurrent connections, with synaptic strength *W*_11_, as well as excites the group of GABAergic interneurons, *x*_2_ (blue), with synaptic strength *W*_12_. In turn, the GABAergic interneurons, *x*_2_, exert a feed-back inhibition on the activity of pyramidal neurons, *x*_1_, with synaptic strength *W*_21_. Further, excitatory glutamatergic projections from pyramidal neurons, *x*_1_, excite the group of dopaminergic neurons, *x*_3_ (pink), with the efficacy *W*_13_. On excitation, the dopaminergic projections from dopaminergic neurons, *x*_3_, release dopamine (dark red) in the DLPFC with the dopamine releasability *W*_34_. The dopamine pool in the DLPFC leads to D1 receptor stimulation and, accordingly, modulates the various parameters involved in cortical dynamics. The two parameters *W*_13_ (efficacy of cortico-mesencephalic projections) and *W*_34_ (dopamine releasability) govern the efficiency of cross-talk between DLPFC and VTA, and are shown as critical tuning knobs which can be easily rotated to different levels to observe their cumulative effect on the whole circuit dynamics.

There are three major dopaminergic pathways present in the central nervous system (CNS) [34]. The nigrostriatal dopaminergic pathway originates from substantia nigra and innervates the basal ganglia. The mesolimbic pathway originates from ventral tegmental area (VTA) and innervates limbic striatum. However, the mesocortical pathway also originates from VTA but innervates the forebrain cortical region (Fig 1A). It is the key source of dopaminergic modulation of PFC through the activation of D1 receptors.

D1 receptors are located on the PFC neurons in great abundance [35] and are mainly situated on the dendritic spines as well as spine shafts of the cortical neurons [36]. These receptors are G-protein-coupled metabotropic receptors whose function is to modulate the activity of various voltage-gated and ligand-gated ionotropic receptors [37]. Activation of D1 receptors in the presence of extracellular dopamine switches on a cascade of intracellular reactions through the activation of second messengers such as cAMP and IP3 [38]. The signaling pathway involves the activation of protein kinases PKA and PKC, further leading to the phosphorylation of various crucial molecules causing their activation, such as dopamine receptor phosphoprotein-32 kDa (DARPP32), and inhibition, such as protein phosphatase-1 (PP1). There also occurs activation of calcium calmodulin kinases (CaMKII and CaMKIV) as well as nuclear transcription factor (CREB) which gives rise to the transcription of various genes including immediate early genes (IEG) and late response genes (LRG). The gene products mainly involve transmembrane proteins and crucial enzymes involved in bringing intrinsic and synaptic plasticity in neurons. The intrinsic plasticity involves changes in the activity of voltage-gated ion channels causing increase in the excitability of neurons.

D1 receptor stimulation in pyramidal neurons leads to decrease in the threshold of depolarizing persistent sodium currents (I_NaP_) and reduction in the inactivating potassium currents (IK^+^) [38, 39]. In the interneurons, increase in the excitability is mainly attributed to the decrease in conductances of the potassium ion channels [40]. Role of D1 receptor activity in enhancing the excitability of cortical pyramidal neurons have been studied in the rat medial PFC [41] as well as in dorsolateral PFC in monkey [42]. The synaptic plasticity involves changes in the conductance of synaptic transmitter-gated ionotropic receptors such as AMPA, NMDA and GABAa receptors. Activation of D1 receptors has been reported to enhance the conductances of NMDA and GABAa receptors whereas there occurs a slight decrease in the AMPA conductance [43–45]. These dopamine-mediated intrinsic and synaptic plasticity play an extremely important role in defining the cortical network activity associated with the working memory task. Iontophoretic studies have shown that NMDA receptors are especially pivotal for the sustained-firing in the cortical network [46]. Any alteration regarding low or high D1 receptor activity makes a debilitating effect on the performance of delayed response tasks [10, 47].

The mathematical description of the prefronto-mesoprefrontal system illustrated here is based on the formalism provided in the original model [28]. This is an extraction from the detailed network investigations involving cortical and mid-brain interactions in the earlier studies performed by Tanaka [29, 30, 48] and involves population-averaged activity of different kinds of neurons. The closed-loop mesocortical circuit consists of two subsystems, DLPFC and midbrain, that are mutually connected. Neurons in the DLPFC are broadly classified into populations of excitatory pyramidal neurons and inhibitory GABAergic interneurons. The excitatory neurons make reciprocal connections with each other together with self-innervations. The excitatory pyramidal neurons also excite the GABAergic interneurons, which exert a feed-back inhibition on the activity of the pyramidal population.

On the arrival of a transient input in the cue interval, the recurrent connections lead to the spreading of firing activity in the excitatory population. Simultaneously, the activity of the excitatory population brings the system of feed-back inhibition into action. Initially, the activity of pyramidal neurons rapidly rises while the GABAergic inhibition is still weak but slowly rising due to the excitation by pyramidal neurons. At a certain level of pyramidal activity, the GABAergic inhibition becomes strong enough to resist any further self-amplification in pyramidal activity. At the same time, the constant self-excitation does not let any decrease in the pyramidal activity to occur due to feed-back inhibition. In this way, the interplay between feed-forward depolarization by pyramidal neurons and feed-back inhibition by the interneurons establishes a sustained firing in the DLPFC. This sustained firing represents the formation of working memory in the delay period.

Moreover, the DLPFC sends out glutamatergic projections to the midbrain where they establish connections with the dopamine neurons. The presence of cortico-mesencephalic projections has been confirmed in various anatomical and physiological studies [49, 50]. Here, the midbrain represents the collection of dopamine nuclei from various mesocortical locations [51]. On excitation, the dopamine neurons release dopamine into the DLPFC through the dopaminergic projections. The excitation of mid-brain during delayed-response working memory tasks have been demonstrated in the *in* *vivo* studies involving primates [52]. This builds up the dopamine pool of the DLPFC which acts as the source of dopamine for the dopaminergic modulation of the DLPFC activity. The dynamical equation outlining the behavior of the prefronto-mesoprefrontal system is described as:

$$\begin{array}{c} \frac{d\vec{x}(t)}{dt}=\vec{f}(\vec{x}) \end{array}$$ (1)

where $\vec{x}{\left(t\right)}^{T}=\{x_{1}(t),x_{2}(t),x_{3}(t),x_{4}(t)\}$, the superscript *T* denotes the transpose of the vector. Here, *x*_1_(*t*) and *x*_2_(*t*) corresponds to the activity of the population of pyramidal neurons and GABAergic interneurons in the DLPFC, respectively. *x*_3_(*t*) represents the activity of the population of dopamine neurons in the mid-brain whereas *x*_4_(*t*) denotes the dopamine content of the DLPFC. Each element of the force vector $\vec{f}$ is given by

$$\begin{array}{c} f_{1}(\vec{x})=-\frac{x_{1}(t)}{\tau_{1}}+W_{11}(d)g(x_{1})-W_{21}g(x_{2}) \end{array}$$ (2a)

$$\begin{array}{c} f_{2}(\vec{x})=-\frac{x_{2}(t)}{\tau_{2}(d)}+W_{12}(d)g(x_{1}) \end{array}$$ (2b)

$$\begin{array}{c} f_{3}(\vec{x})=-\frac{x_{3}(t)}{\tau_{3}}+W_{13}g(x_{1}) \end{array}$$ (2c)

$$\begin{array}{c} f_{4}(\vec{x})=-\frac{x_{4}(t)}{\tau_{4}}+W_{34}g(x_{3}) \end{array}$$ (2d)

The first term on the right-hand side of Eq (2a), (2b) and (2c) denotes the excitability of the excitatory, inhibitory and dopaminergic neuronal populations, respectively, defined by their specific time constants *τ*_1_, *τ*_2_ and *τ*_3_. A larger time constant implies greater excitability of the constituent neurons. The second term in Eq (2a), (2b) and (2c) represents the recurrent excitation of the pyramidal population, excitation of interneurons and dopaminergic neurons, respectively, with the corresponding synaptic efficacies *W*_11_, *W*_12_ and *W*_13_. The last term in Eq (2a) denotes the inhibition of pyramidal neurons by the interneuron activity with synaptic efficacy *W*_21_. For simplicity, the inhibitory connections among interneurons have not been considered in the original model by Tanaka [28]. The presence of inhibitory connections among interneurons affects the resultant ratio of excitatory activity performed by pyramidal neurons and inhibitory activity performed by interneurons on the pyramidal activity in the cortical region. Therefore, the putative effects of such connections on the cortical dynamics have already been adjusted in the baseline magnitudes of the excitatory and inhibitory synaptic efficacies present in the model. Further, the first and second terms in Eq (2d) denote the absorption/uptake with time constant *τ*_4_ and release of dopamine from dopaminergic afferents with releasability parameter *W*_34_, respectively. A schematic diagram describing these interactions is provided in Fig 1B. The variable *d* represents the level of D1 receptor activation in the presence of dopamine and is related to the dopamine content of the DLPFC as

$$\begin{array}{c} d=d_{\max}g(x_{4}) \end{array}$$ (3)

Here, *d*_*max*_ is the maximum level upto which the D1 receptors can be activated.

The activity of the variables does not increase indefinitely, but rather saturates at a finite value. Many possible functions exist that capture this behavior. One such function *g*(*x*_*i*_) has been employed in the model.

$$\begin{array}{c} g(x_{i})=\{\begin{array}{c} \tanh(ax_{i}),x_{i}(t)\geq 0\\ 0,x_{i}(t)<0 \end{array}\},(i=1,2,3,4) \end{array}$$ (4)

The dopaminergic modulations of the synaptic strengths *W*_*ij*_ in the DLPFC are modeled as $W_{11}=W_{11}^{*}(0.12d+0.68)$ and $W_{12}=W_{12}^{*}(0.12d+0.68)$. Accordingly, the synaptic strengths increase with the rise in D1 receptor stimulation and, thus, manifests into the synaptic plasticity [38]. Moreover, the time constant *τ*_2_ is given by $\tau_{2}=\tau_{2}^{*}\left(0.24z+0.26\right)$. The decrease in time constant *τ*_2_ with rise in D1 receptor stimulation denotes the increase in the excitability of inhibitory GABAergic interneurons due to the associated decrease in potassium channel conductance [40]. $W_{11}^{*},W_{12}^{*}$ and $\tau_{2}^{*}$ are the basal values of the respective parameters.

Here, the cue, phasic and tonic input currents have not been introduced, which are present in the original model. These currents mainly determine the temporal dynamics of the system keeping the equilibrium configuration of the system intact. The present study is intended to investigate the steady state properties of the circuit (in the absence and presence of noise) and, therefore, these currents can be reasonably ignored.

### Critical parameters in the model

The two circuit parameters *W*_13_ (efficacy of corticomesencephalic glutamatergic transmission) and *W*_34_ (dopamine releasability from dopaminergic afferents) are considered very crucial in the model dynamics as they control the efficiency of the cross-talk between the DLPFC and the midbrain in the circuit. They act like critical tuning knobs (as shown in Fig 1B) in the model which can be easily rotated to different levels and, hence, these parameters may be altered in several ways to see their subsequent effects on the circuit dynamics. These alterations may possibly be involved in various complex clinical situations. However, a definitive and fruitful understanding of the system’s behavior can be obtained only when a systematic approach towards alterations in these parameters is adopted. With this intention, the study considers the setup where the dopamine releasability *W*_34_ is varied while holding the corticomesencephalic efficacy *W*_13_ fixed at its normal value i.e. 100% efficacy.

The importance of this setup lies in the fact that it allows to espy purely the effect of dopaminergic alterations on the system’s response when rest of the circuit’s cytoarchitecture and function is intact. Alterations in dopamine transmission have been shown in brain imaging studies with schizophrenic patients [53]. The postmortem study performed by Akil and Lewis [54] have shown reduction in dopaminergic projection innervating DLPFC in schizophrenic patients. Abnormality in the functioning of midbrain dopamine neurons also occur under schizophrenia [16, 55]. Similarly, alterations in dopaminergic response has also been reported in ageing conditions [11, 56]. Even the dopamine elevating drugs such as FG7142, leading to acute stress conditions, also involve heightened dopaminergic release in DLPFC [22].

When the level of dopamine release response, while its variation, is adjusted to its normal value, the setup captures the normal functioning state of the mesocortical circuit involved in working memory activity under healthy conditions. This reflects an additional benefit of the setup as it provides a reference point for comparison with abnormal states of the system associated with abnormal levels of dopamine release response. Altogether, the present setup for variation in crucial parameters may serve as a building block to a more integrated and complex description of circuit dynamics underlying the normal as well as clinical conditions.

### Equilibrium analysis of the dynamical system

The global equilibrium state of the system is given by

$$\begin{array}{c} \frac{d\vec{x}(t)}{dt}=\vec{0} \end{array}$$ (5a)

which yields

$$\begin{array}{c} f(x^{*})=0 \end{array}$$ (5b)

For notational convenience, the superscript * is dropped to read as

$$\begin{array}{c} f(x^{*})=f(x)=0 \end{array}$$ (5c)

**Fig 2:**
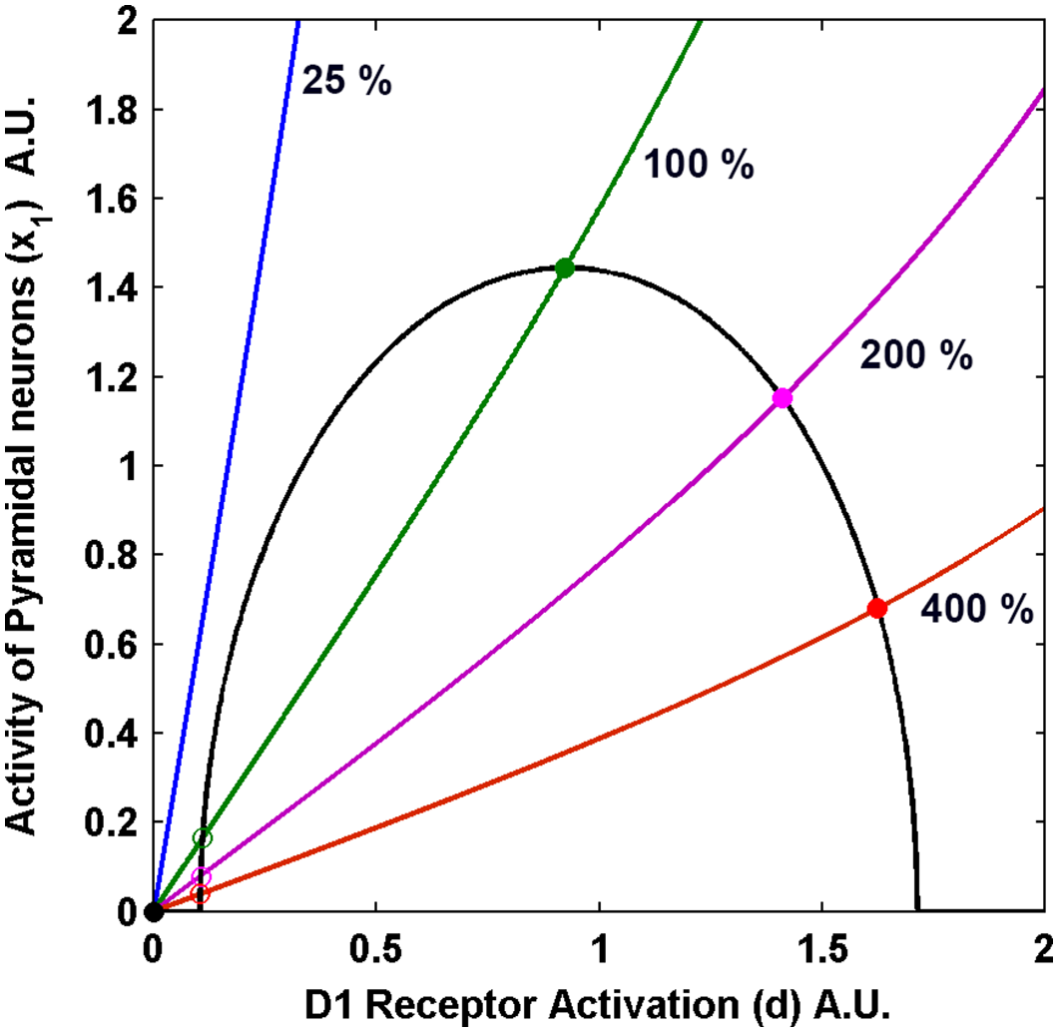
Nullcline Plots of the Pyramidal Neurons Activity, *x*_1_ versus D1 Receptor Activation, *d* for different values of dopamine releasability, *W*_34_. *W*_34_ is indicated by the % values relative to 0.36. The solid (open) circles represent stable (unstable) fixed points. The intersection points of the curves are the points of global equilibrium of the system, for that particular *W*_34_. The parameters used here are $\tau_{1}=20,\tau_{2}^{*}=6.8,\tau_{3}=10,\tau_{4}=800$ and $W_{11}^{*}=0.5588$, $W_{12}^{*}=0.786$, *W*_13_ = 0.023, *W*_21_ = 0.339, *a* = 0.15.

**Fig 3:**
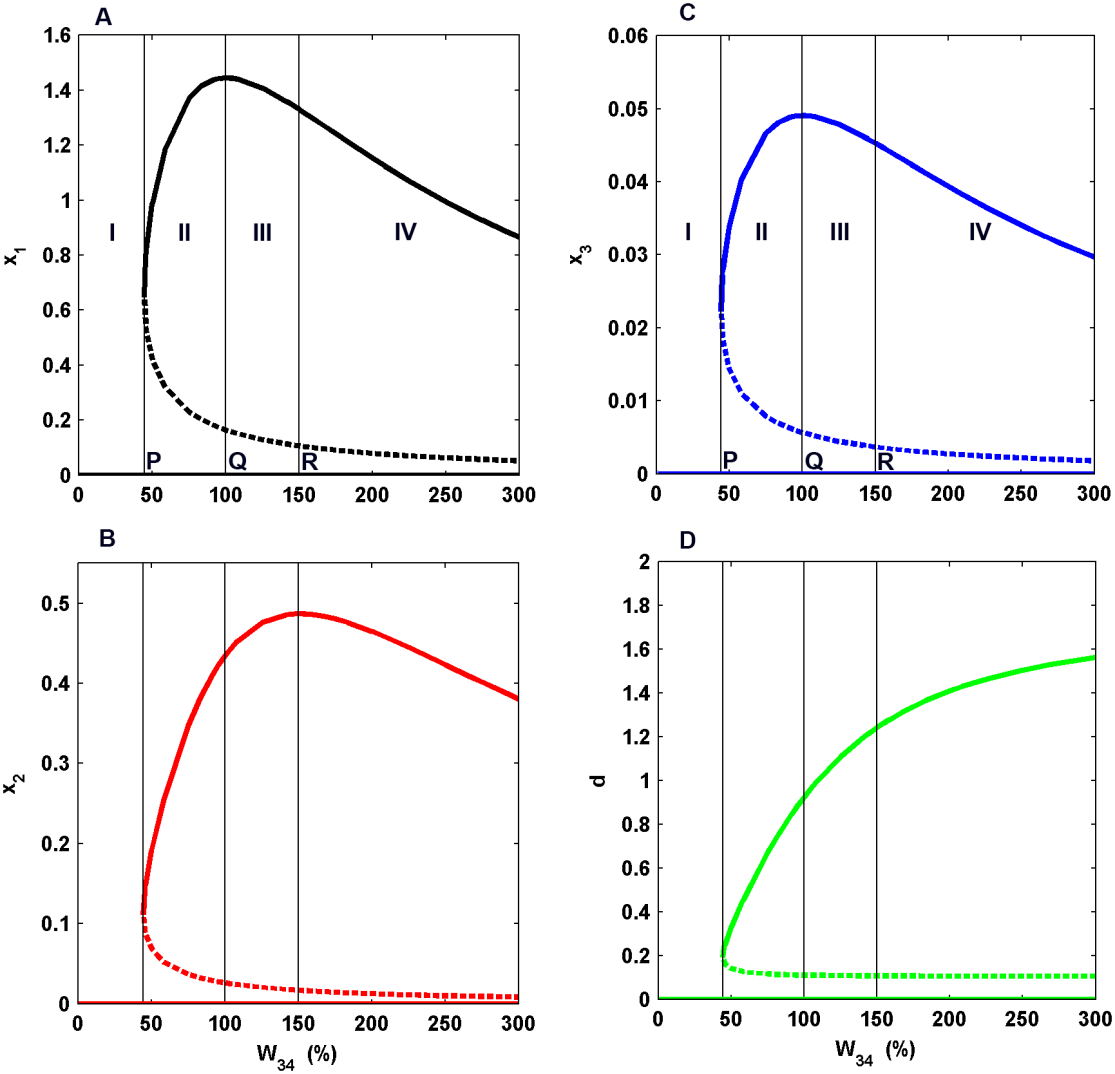
Bifurcation Diagrams. These curves depict the equilibrium activity of the pyramidal neurons (A), inhibitory neurons (B), dopaminergic neurons (C) and D1 receptor activation (D) versus the bifurcation parameter *W*_34_. The solid (dotted) lines portray stable (unstable) states of the system. The curves are partitioned into four regions I, II, III and IV. Point P, Q and R are the boundary points of these regions. Point P is the point at which bistability appears in the system. Point Q is the point of maximum equilibrium activity of excitatory neurons and Point R is the point of maximum equilibrium activity of interneurons.

Using Eq (5c), the nullcline plots of *x*_1_ and *x*_4_ in the *x*_1_-*x*_4_ state space are obtained and shown in Fig 2. The intersection points of these nullclines signify the global equilibrium state of the system. The intersection points marked as open and solid circles are the unstable and stable fixed points, respectively. From this study, the bifurcation plots of the variables with dopamine releasability *W*_34_ as the bifurcation parameter are obtained as shown in Fig 3.

### Stochastic Model: Circuit Dynamics in Noisy environment

The dynamics of excitatory as well as inhibitory populations in the DLPFC and dopamine neurons in the midbrain are constantly affected by two major sources of noise viz. intrinsic noise in various voltage-gated (Na^+^ and K^+^ ion channels) as well as ligand-gated receptors (AMPA and NMDA receptor at excitatory and GABA receptors at inhibitory synapses) and the extrinsic noise due to surrounding neuronal activities in the brain [31, 33]. Moreover, fluctuations in the dynamically varying dopamine content of the DLPFC arises from the stochastic binding of dopamine by dopamine receptors located on the local cortical neurons and its absorption by reuptake transporters located on dopaminergic afferents and the accessory glial cells. To capture the fluctuations in the circuit dynamics collectively arising from these sources of noise, a stochastic white noise force vector $\vec{\zeta }(t)$ is introduced into the dynamical system.

$$\begin{array}{c} \frac{d\vec{x}(t)}{dt}=\vec{f}(\vec{x})+\vec{\zeta }(t) \end{array}$$ (6)

Since the goal of the present investigation is to look into the steady state properties of the mesocortical circuit associated with the delay-period, the timescale of the macroscopic dynamics is of the order of seconds, which is typical of the delay interval in the experimental studies involving delayed-response tasks with primates [8, 10]. However, the time scale of random fluctuations in the conductances of different ion channels as well as synaptic receptors [33] and the fluctuations in dopamine content of DLPFC due to receptor binding and reuptake transporters is of the order of milliseconds and is much smaller than that of the macroscopic dynamics. Therefore, the stochastic noise is assumed to be delta-time autocorrelated which leads to an independently distributed random variables in the time series devoid of any temporal structure [33].

Moreover, there are several different kinds of noise sources which are being collectively considered in the present stochastic framework. These noise sources work at relatively different time scales. Therefore, by the central limit theorem, the combined effect of these noise leads to Gaussian noise process linearly introduced (additive noise) into the deterministic dynamical system [33]. More formally, the Langevin equations are rewritten as

$$\begin{array}{c} dx_{1}=f_{1}(\vec{x})dt+\sigma_{1}dW_{1} \end{array}$$ (6a)

$$\begin{array}{c} dx_{2}=f_{2}(\vec{x})dt+\sigma_{2}dW_{2} \end{array}$$ (6b)

$$\begin{array}{c} dx_{3}=f_{3}(\vec{x})dt+\sigma_{3}dW_{3} \end{array}$$ (6c)

$$\begin{array}{c} dx_{4}=f_{4}(\vec{x})dt+\sigma_{4}dW_{4} \end{array}$$ (6d)

The functions $f_{i}(\vec{x})$ are the same as described in ‘The model’ section. {*dW*_*i*_(*t*), *t* ≥ 0}, (*i* = 1, 2, 3, 4) define the Weiner process increment. It is also assumed that these increments are independent of each other. The noise terms represent additive noise with strength, *σ*_*i*_, (*i* = 1, 2, 3, 4).

Earlier studies regarding mean-field analysis of network dynamics, such as by Amit and Brunel [57]; Brunel and Sergi [58]; Brunel and Wang [23]; Loh, Rolls and Deco [27], have predominantly considered Poissonian noise which originates from random firings of neurons in a finite-sized cortical network. However, the present model of noise attempts to consider a bigger repertoire of noise elements, including the poissonian noise in spiking cortical network. Under these circumstances, explicit modeling of various noise terms may yield an unwieldy dynamical structure which may prove futile in providing any clear insight into the system’s behavior. This necessitates the conception of a minimal structure of noise which can serve the demands of the present investigation. As evident, the structure of noise modeled here is minimal and effective, in the sense that least number of noise terms are required to essentially capture the effects of various noise prevailing in real scenario.

Another remarkable fact to be noted is that the expectation of the system’s response under noisy perturbation resembles its deterministic response in the present framework of noise. In other words, the additive noise structure preserves the signal profile of the circuit dynamics. This allows the study of the system’s stability and robustness along the lines of its signal counterpart.

The combined effect of the deterministic and stochastic dynamics decides the path of the trajectories leading to either of the attractor states. Noise causes a spread out of the initial states of the system in the temporal dynamics leading to a statistical distribution of the various variables in the state space. In this regard, the steady state distributions of the variables are helpful in gaining insight into the properties of the circuit dynamics under noisy environment. The function $P_{st}(\vec{x})$ describes the steady state distribution which is directly linked to the global potential landscape in the multidimensional state space. Higher the probability $P_{st}(\vec{x})$ of a configuration, lesser is the potential associated with that configuration and, hence, the more stable it is. The state of the system associated with the maximum $P_{st}(\vec{x})$ is the most stable and its corresponding global potential minimum.

### Simulation Procedure

A corresponding multidimensional Fokker-Planck equation can be obtained for Eq (6a)–(6d), describing the statistical temporal-evolution of the variables distributions. The solution of such a multidimensional partial differential equation (PDE) is quite challenging and thus one resorts to simulation procedure. Monte Carlo Simulation based on Euler Maruyama numerical scheme with fixed time-step [59] is employed to obtain the evolution of the time trajectories of the variables. To simulate a trajectory using the Euler Maruyama scheme for a given time discretization the initial values *x*_*i*_(*t* = 0) = *x*_0_ is the starting point and recursively utilizing Eq (7a)–(7d) one proceeds to obtain the next value.

$$\begin{array}{c} x_{1}(t+\Delta t)=x_{1}(t)+f_{1}(\vec{x})\Delta t+\sigma_{1}Z_{1}\sqrt{\Delta t} \end{array}$$ (7a)

$$\begin{array}{c} x_{2}(t+\Delta t)=x_{2}(t)+f_{2}(\vec{x})\Delta t+\sigma_{2}Z_{2}\sqrt{\Delta t} \end{array}$$ (7b)

$$\begin{array}{c} x_{3}(t+\Delta t)=x_{3}(t)+f_{3}(\vec{x})\Delta t+\sigma_{3}Z_{3}\sqrt{\Delta t} \end{array}$$ (7c)

$$\begin{array}{c} x_{4}(t+\Delta t)=x_{4}(t)+f_{4}(\vec{x})\Delta t+\sigma_{4}Z_{4}\sqrt{\Delta t} \end{array}$$ (7d)

Here, *Z*_1_, *Z*_2_, *Z*_3_, *Z*_4_ represent the Gaussian random variables with zero mean and unit variance i.e. *Z*_*i*_ ∼ *N*(0, 1), (*i* = 1, 2, 3, 4).

The above procedure is carried out for different values of the circuit parameter, *W*_34_. In the simulation procedure the unstable fixed points in the state space are considered as the initial state of the system, *x*_0_. Since the individual numerical solutions differ significantly from the true solutions, a statistical analysis is an essential consideration. Also the Monte Carlo procedure has the relative error *O*(*N* ^−1/2^) [60]. In this study, the steady state probability distribution $P_{st}(\vec{x})$ is obtained after 1,20,000 time steps for a fixed time-step Δ*t* = 0.001 and over an ensemble of *N* = 10^6^ realizations. The code used to simulate the stochastic mesocortical dynamics will be posted on ModelDB(https://senselab.med.yale.edu/ModelDB/).

From these steady state distributions, the global potential landscape of the system can be obtained using

$$\begin{array}{c} U(\vec{x})∼-\ln(P_{st}(\vec{x})) \end{array}$$ (8)

## Results

### Deterministic dynamics of the circuit

To facilitate the analysis, the entire range of *W*_34_ has been divided into four zones (zone I, II, III and IV) separated by the points P, Q and R (Fig 3). The lower stable fixed point in the four dimensional state space signifies the spontaneous-activity state or basal-activity state of the system where rare firing-events occur. Once an external stimulus arrives in the cue interval, it sets the system into action. The higher stable fixed point in the state space over the region of bistability signifies the densely firing active state of the system where sustained firings in the DLPFC are established. For convenience, this state is referred as the sustained-firing state of the system. These sustained firings represent the formation of a working memory under the present dynamical framework.

A monostable region exists for the lower values of the dopamine releasability (zone I) where no sustained firing can be achieved. The point P signifies the critical dopamine releasability at and above which the system makes a transition from the state of basal activity to the state of sustained firing. The unstable state sets a threshold which when overcome by the system in the presence of cue input leads to the formation of a working memory, following the deterministic setting of the model. In the bifurcation plot of *x*_1_ (Fig 3A), magnitude of the variable *x*_1_ at the higher stable state/ sustained-firing state alludes to the intensity of sustained-firing underlying a working memory representation in the real scenario during delay period. In a similar manner, magnitudes of the variables *x*_2_, *x*_3_ and *d* at the higher stable state (Fig 3B-3D) suggest the activities of inhibitory GABAergic interneurons, mid-brain dopaminergic neurons and D1 receptor activation, respectively, contributing to the establishment of sustained firing during delay period.

Increase in the dopamine releasability markedly affects the D1 receptor activity, which further modulates the activities of pyramidal and interneuron populations. In zone II, the intensity of the equilibrium pyramidal activity is seen to rise with the rise in D1 receptor activity (Fig 3A and 3D). At point Q, the pyramidal activity reaches its maximum. However, the equilibrium interneuron activity is still in its increasing phase (zone II and III) and reaches its maximum at point R (Fig 3B). This observation bears similarity with the mechanism of dopaminergic modulation of DLPFC activity and working memory suggested by Goldman-Rakic [21]. However, in zone IV, the equilibrium intensities of the pyramidal as well as interneuron activities both decrease together.

### Stochastic dynamics of the circuit

**Fig 4:**
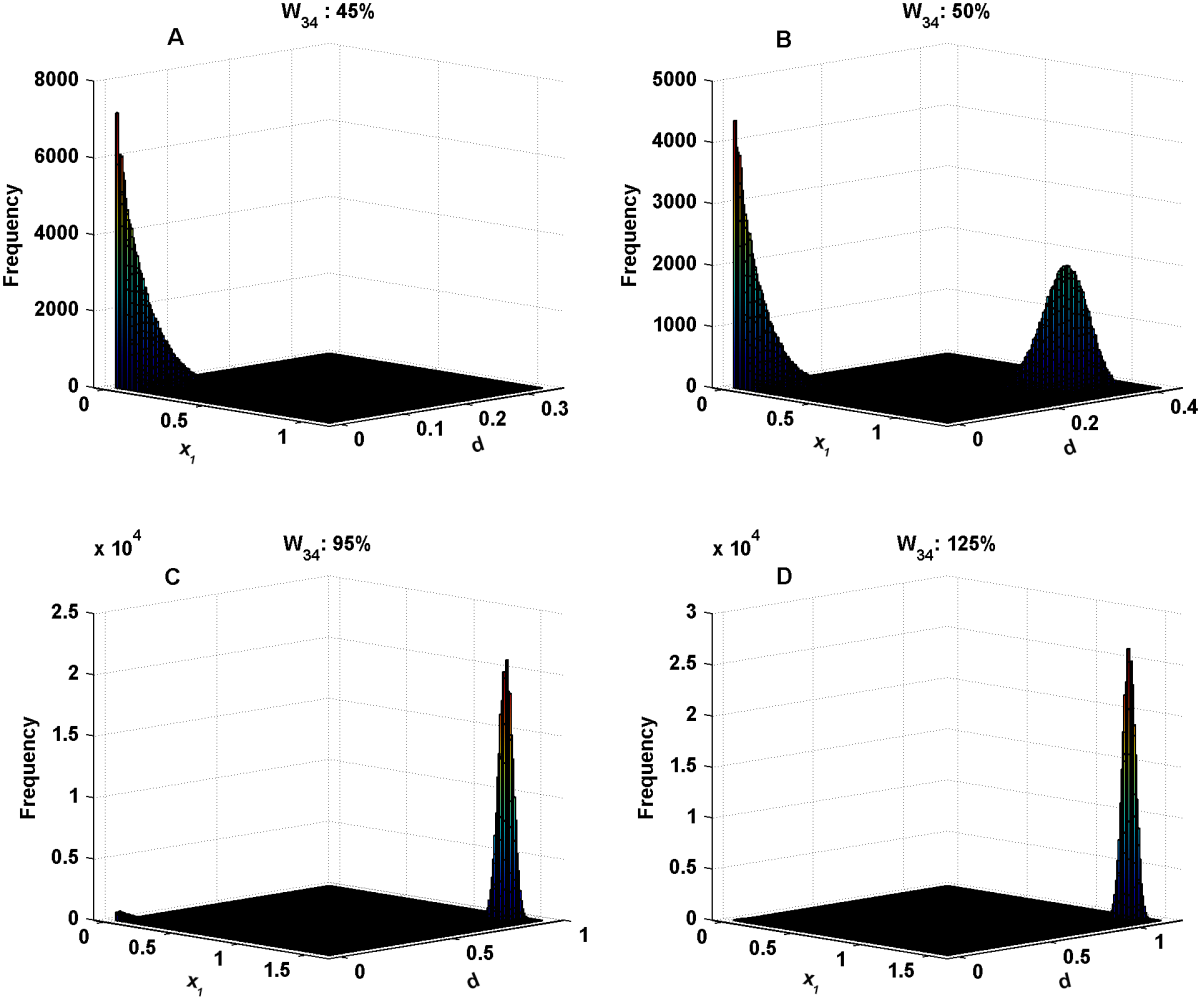
Steady state frequency distributions for different values of dopamine releasability, *W*_34_. These distributions are obtained by simulating Eqs (7a)–(7d). With increasing *W*_34_, the curves exhibit a gradual transition of relative sample distributions from lower to higher stable state. Additive noise strengths used here are *σ*_1_ = 0.05, *σ*_2_ = 0.01, *σ*_3_ = 0.001, *σ*_4_ = 0.05, estimated in accordance with the scales of magnitude acquired by the different variables in the circuit dynamics.

From the numerical simulation of the Langevin Eq (7a)–(7d), the steady state distributions of the variables are obtained. Since it is difficult to demonstrate pictorially the distribution of the variables in the multidimensional state space, it is convenient to show it only for the two dimensional *x*_1_-*d* state space (Fig 4). In zone I, the only stable state that exists is the lower stable state where the system remains in the spontaneous-activity mode and, hence, does not provide any significant observation in the presence of noise. For this reason, this region is excluded from examination in the study. In the region of bistability, it is seen that the lower stable state in zone II (close to P) is much more sample-populated than the corresponding higher stable state in the histogram plot (Fig 4A). Moving towards higher dopamine releasability *W*_34_, the higher stable state is relatively more sample-populated in comparison to the corresponding lower stable state (Fig 4C-4E). It is evident that there occurs a gradual shift in the profile of relative sample distributions from the lower to higher stable state with the increase in dopamine releasability. And in Fig 4B, the distribution profile demonstrates an almost intermediate of this shift. From these steady state distributions, the global potential landscape of the circuit is procured.

**Fig 5:**
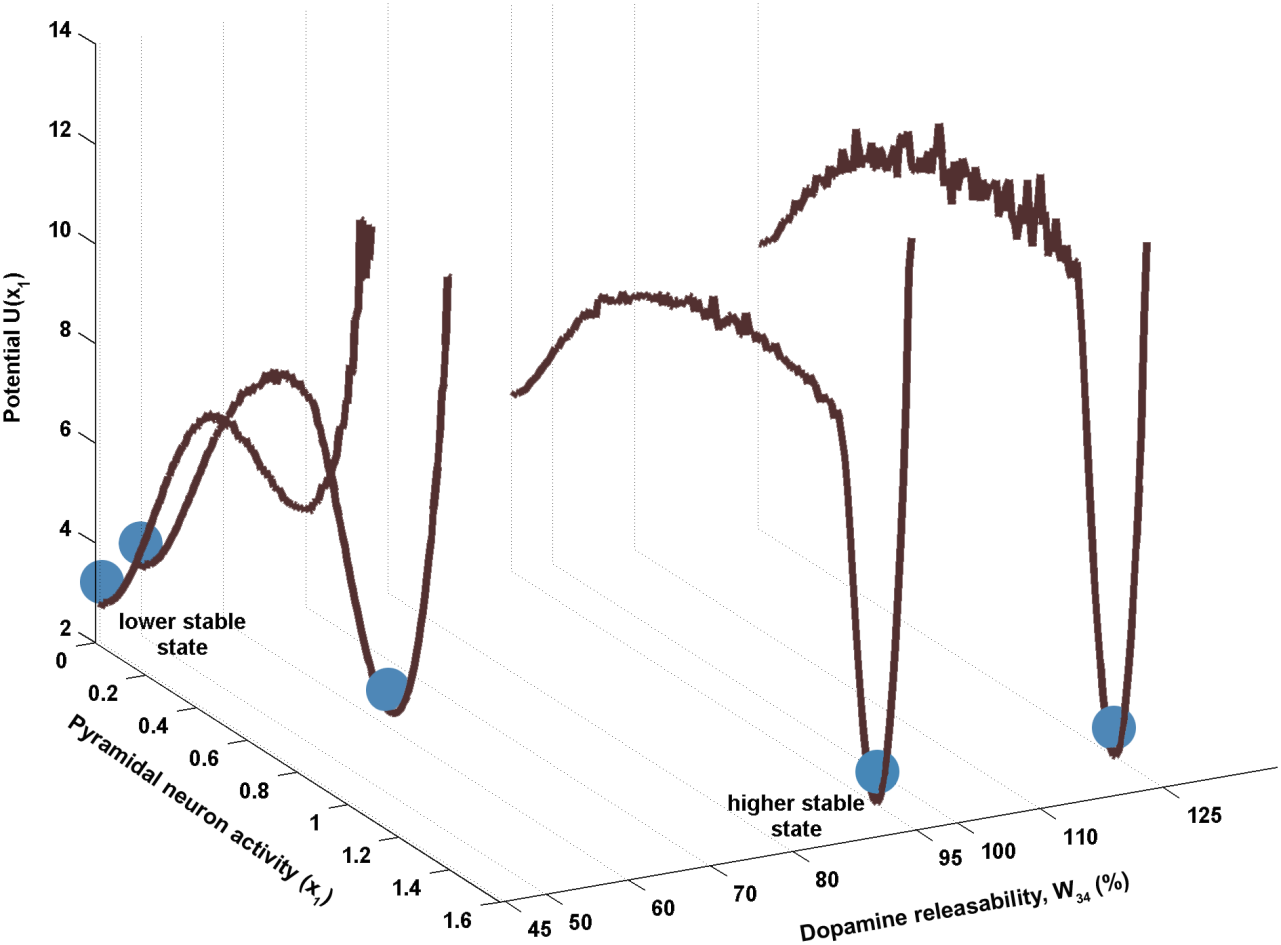
Global potential landscape w.r.t the pyramidal neuron activity *x*_1_, for different values of DA releasability *W*_34_. The profiles are obtained from the steady state distributions in variable *x*_1_ for different values of *W*_34_. Here, the lower stable state represents the spontaneous-activity state and the higher stable state represents the sustained-firing state (working memory state) of the system. The system has been depicted by a ball sitting in the potential wells belonging to the different activity states. At low *W*_34_, the system (ball) prefers to rest in the well associated with the lower stable state as it is relatively more robust in comparison to its corresponding higher stable state. However, with the increase in *W*_34_, the higher stable state gradually becomes more robust relative to the corresponding lower stable state.

The global potential landscape with respect to the variable *x*_1_ is demonstrated for a few selected values of *W*_34_ for better illustration(Fig 5). It is seen that the potential surface exhibits a rugged behavior. For lower values of the dopamine releasability (zone II close to P), the lower stable state is more stable than the corresponding higher stable state in the sense that the former has a significantly lower potential in comparison to the latter. This implies that the sustained firings at the higher stable states formed in this region during the delay period are prone to collapse to the lower stable states in the presence of noise. Moving further towards point Q, a situation is encountered where both states have almost equal potentials but are separated by a strong potential barrier. However, at higher values of the dopamine releasability (zone II (near Q), III and IV), the higher stable states are more stable than the lower stable states indicating that the sustained-firing states of the system in these regions are substantially resistant against collapse to the spontaneous-activity state of the system under noisy perturbations. The strength of a stable state of the system in the region of bistability, to retain itself in that state in the presence of noisy fluctuations will be referred to as the robustness of that state. Accordingly, it may be asserted that the robustness of the sustained-firing state of the system is significantly higher at higher levels of dopamine releasability in comparison to that at lower dopamine releasability.

**Fig 6:**
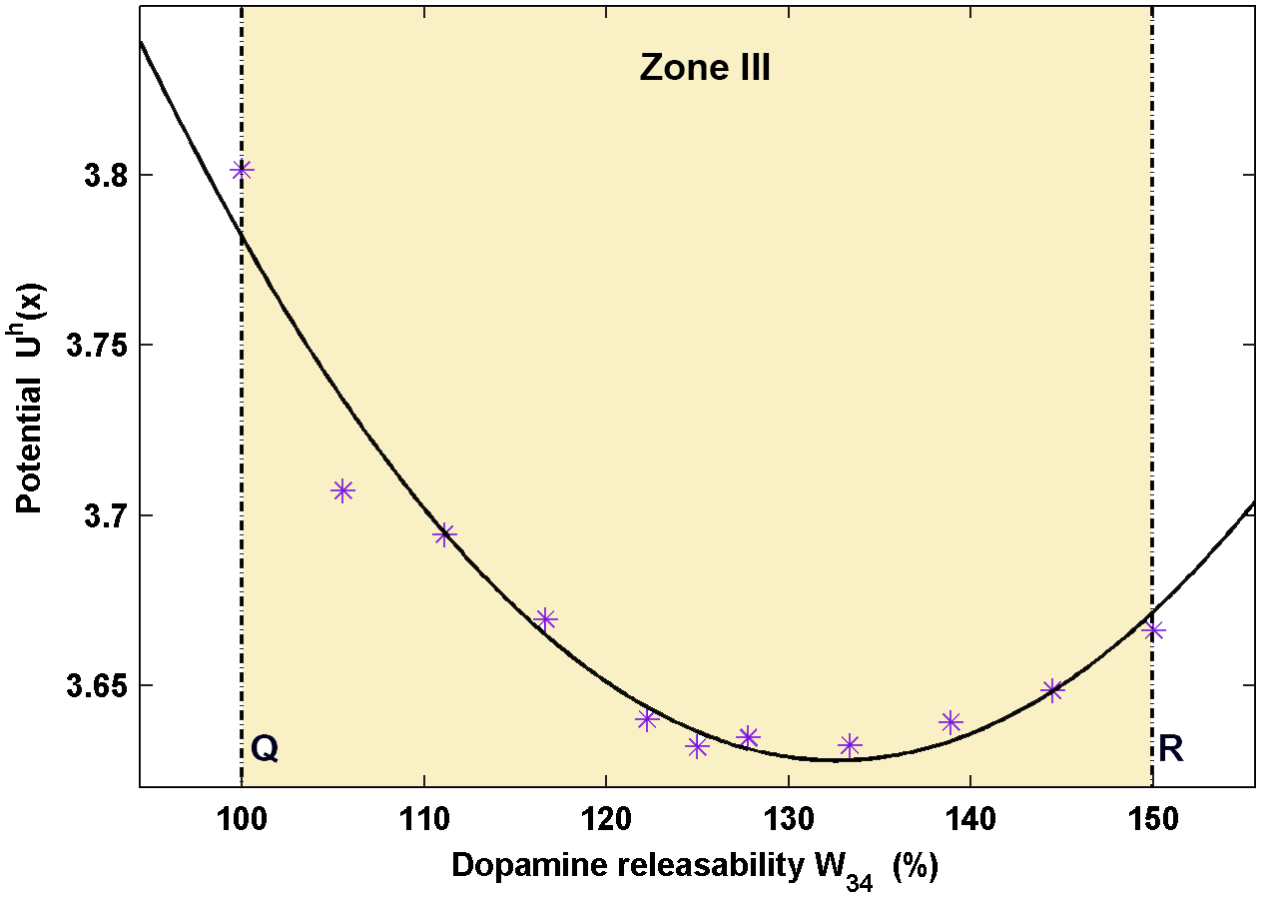
Profile of variation in potential of higher stable state with change in dopamine releasability *W*_34_. The points shown in asterisks denote the potentials of higher stable states for the respective dopamine releasability, *W*_34_ in zone III obtained from the global potential landscape. The solid curve is a fitting to the data. The potential is minimum in the region between the points Q and R of maximum equilibrium excitatory and inhibitory activities, respectively. The point of minima represents the maximum robustness that the circuit dynamics may achieve during the delay period. This further suggests the existence of a subtle ratio of the equilibrium excitatory to inhibitory activity which fine tunes the circuit’s robustness and leads to an inverted-U shaped profile of the robustness of higher stable states during the delay period.

It is further essential to view closely how the circuit’s robustness varies among the higher stable states with the change in dopamine releasability, in the region of sufficiently high level of D1 receptor activity. For this, the values of the potential $U^{h}\left(\vec{x}\right)$ of the higher stable fixed points obtained from the global potential landscape in the zones II (near Q), III and IV are plotted with respect to the parameter *W*_34_ (Fig 6). Here, the superscript *h* denotes the higher stable state of the system. It is observed that with the increase in the dopamine releasability, the potential of the fixed point first decreases and reaches a minimum. A further increase in the dopamine releasability leads to a rise in the potential of the higher stable fixed point.

Since robustness of the higher stable state is inversely related to its potential in the global landscape, Figs 5 and 6 together suggest that increase in the dopamine releasability, in turn, the D1 receptor activity, initially leads to an increase in the robustness of higher stable state of the system, but a further increase causes reduction in the robustness. However, it must be kept in mind that the robustness of the higher stable state still remains much higher than that of the corresponding lower stable state in the region of decreasing robustness at very high dopamine releasability. At a particular value of *W*_34_, there exists the most robust configuration of the system.

### Signal-to-noise ratio (SNR)

The above two subsections discuss the signal and noise aspects of the circuit dynamics, separately. Here, we compile these two aspects together and explain signal-to-noise ratio. It signifies the relative strength of a signal against its noisy fluctuations. It is given by,

$$\begin{array}{c} SNR=\frac{E[signal]}{\sqrt{Var[signal]}} \end{array}$$ (9)

**Fig 7:**
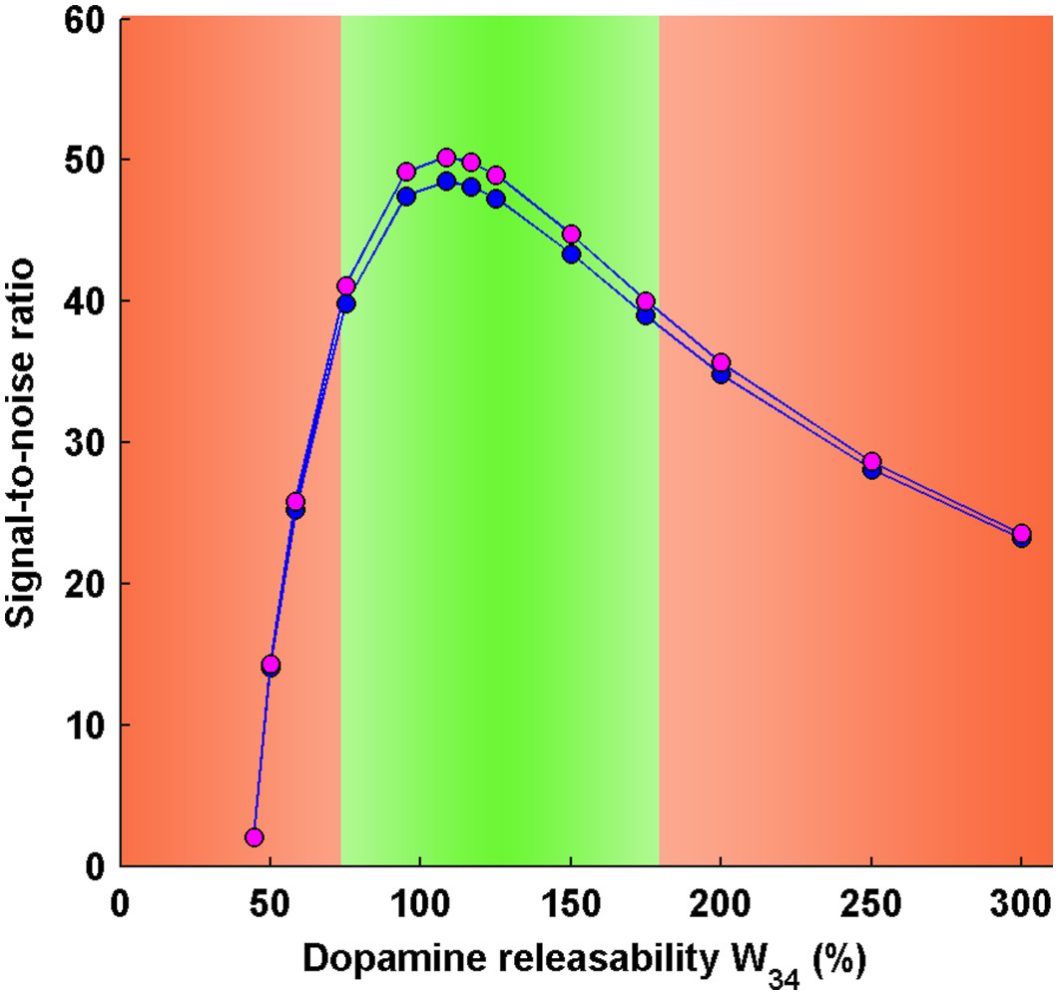
Signal-to-noise ratio (SNR) in pyramidal and midbrain activities in the working memory state during delay interval. The SNR profiles of pyramidal and midbrain activities exhibit an inverted-U shaped profile similar to the signal profile seen in the bifurcation diagram. The SNR profiles are consistent with the error bar plots of pyramidal and midbrain activities, respectively, during delay interval. The optimal region (green zone) is associated with maximum signal-to-noise ratio and signifies establishment of efficient working memory during delay interval. At abnormal levels of dopamine releasability *W*_34_ (right and left, red zone), there occurs a significant decline in the signal-to-noise ratio which may lead to working memory impairment.

**Fig 8:**
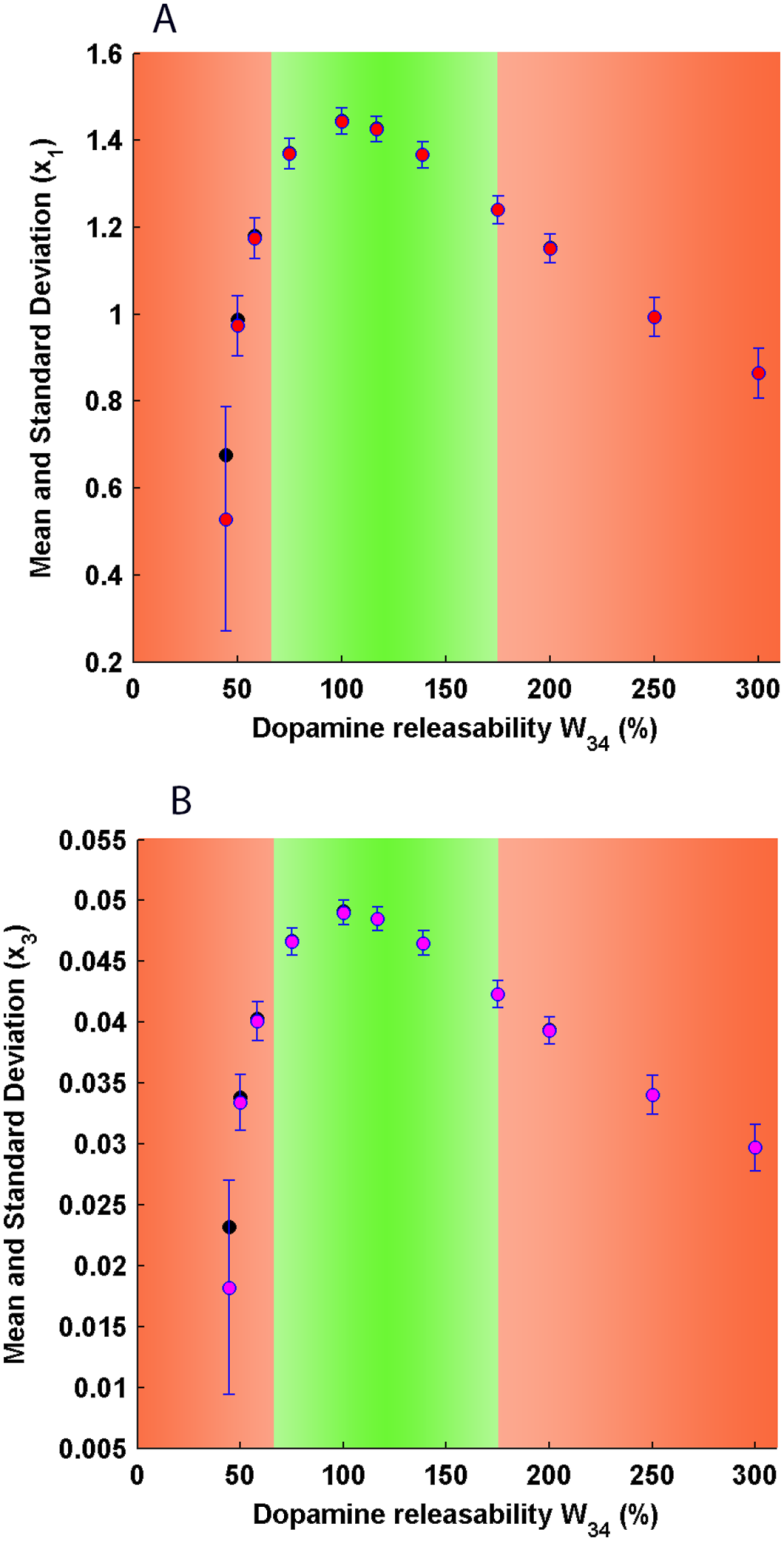
Error bar plots of pyramidal (A) and dopaminergic midbrain (B) activity under stochastic mesocortical dynamics during delay interval. The solid red (magenta) circles represent working memory-associated mean pyramidal (midbrain) activities and the blue error bars denote standard deviations around the mean activities at different dopamine releasability *W*_34_, in the presence of noisy circuit dynamics. The solid black circles represent pyramidal (midbrain) activities obtained from deterministic mesocortical dynamics and constitutes the signal profile. In the optimal region (green zone), the mean pyramidal (midbrain) activity is associated with least noisy fluctuations and coincides with the pyramidal (midbrain) activity of the signal profile. In the region of low dopamine releasability (left, red zone), the pyramidal (midbrain) activity shows dramatic fluctuations such that the mean activity significantly deviates away from the sustained-firing state and shifts towards the spontaneous-activity state. In the region of high dopamine releasability (right, red zone), there does not occur a noticeable deviation of the mean pyramidal (midbrain) activity from the sustained firing state, but, indeed contains significant fluctuations as compared to the optimal region. As evident, the pyramidal and midbrain activity demonstrates a tightly-linked response with regard to their relative noise content.

In the present study, the signal-to-noise ratio is obtained from the sample probability distribution in steady state. The signal-to-noise ratio of pyramidal activity in the DLPFC during the working memory state of the circuit (higher stable state), under different conditions of dopamine releasability, is shown in Fig 7. For the same, the expectation of the activity (signal) and the standard deviation around the mean activity are also shown in Fig 8. It is evident that the signal-to-noise ratio in DLPFC firing is maximal near 100% dopamine releasability, *W*_34_. As one moves away from this region, the ratio decreases but in an asymmetric manner where the decrease is steeper towards lesser dopamine releasability.

A similar observation is made through the error-bar plot (Fig 8A). In close proximity to 100% *W*_34_, the mean activity coincides with the signal obtained from the deterministic analysis and contains least fluctuations. However, while moving towards lesser dopamine releasability, a sharp deviation of the mean activity from the deterministically estimated signal is observed along with dramatically increasing fluctuations. Notably, this deviation occurs towards the spontaneous state of the system (lower stable state). On the other hand, towards higher dopamine releasability, one does not observe noticeable deviations of the mean activity from the deterministically estimated signal but does observe a gradual increase in fluctuations in the activity.

Further, the signal-to-noise ratio in the firing (activity) of dopaminergic neurons in the midbrain, during working memory state, is also shown together with that of pyramidal activity in the DLPFC (Fig 7). Both share a similar profile with maxima around 100% *W*_34_. When the error-bar plot of midbrain activity (Fig 8B) is compared with that of pyramidal activity (Fig 8A), one again observes shared features in their responses. This demonstrates a tightly linked response of cortical and midbrain activity with regard to its noise content. The mechanistic reason behind this observation relies on the intricately coupled dynamics of various compartments of the circuit which not only allows the flow of signal but also noise along the whole circuit pathway. Therefore, this implies that poor activity in DLPFC hampers the quality of excitatory transmission along the glutamatergic projection involved in engaging the midbrain activity and engenders an equally poor midbrain response.

## Discussion

### D1 Receptor activity governs global robustness of the circuit dynamics

It may be noted that in the model, the effect of dopamine releasability on the circuit dynamics is primarily through its effect on the level of D1 receptor activation. This is so because the D1 receptor activity is critically involved in shaping the reciprocal interactions among the DLPFC pyramidal neurons and inhibitory neurons. In turn, it affects the excitation of dopamine neurons in the midbrain and sets up the dynamical behavior of the overall circuit as well as its steady state properties. Moreover, the higher steady state value of D1 receptor activity *d*, monotonically increases with the rise in dopamine releasability (Fig 3D). Therefore, in light of these facts, it is realised that variation in the robustness of the stable states of the system observed with the rise in dopamine releasability is actually being governed by the concomitant rise in D1 receptor activity. It is further observed that zones II (near Q), III and IV are associated with relatively high level of equilibrium D1 receptor activity in contrast to zone II (near P) where it is significantly low (Fig 3D). Coincidentally, it is also observed that the higher stable state is more robust in comparison to the corresponding lower stable state at any given dopamine releasability in zones II (near Q), III and IV whereas the reverse is true in zone II (near P) (Fig 5). Therefore, these observations together indicate that a sufficiently high level of equilibrium D1 receptor activity favors the sustained-firing state over the basal-activity state of the system during the delay period. This is an important finding as it demonstrates that the formation of a robust working memory under natural conditions of a noise-perturbed circuit dynamics requires a sufficient level of D1 receptor activity so that the sustained firing established in the DLPFC during the delay period could retain itself against the disrupting effect of background noise.

A similar finding has been reported in the computational studies performed by Durstewitz et al. [25, 26, 61] where leaky integrate-and-fire model and compartmental models were employed to investigate the robustness of working memory-associated neural representations under the effect of D1 receptor activity during delay interval. It was shown that high levels of D1 receptor activity make the sustained firing activity strongly robust against distractions during the delay period. Even in the computational studies performed by Loh, Rolls and Deco [27, 62] employing integrate-and-fire neural attractor networks, a similar increase in the robustness of sustained firing state was demonstrated with rise in the NMDA and GABA conductances, which may be realized under increase in D1 receptor stimulation. They also provided an energy landscape description for the robustness of the different states (spontaneous and persistent) associated with cortical activity during delay interval. However, the findings of the present study serves its own novelty in many ways.

One common thing in all the earlier studies is that the presence of dopamine was not explicitly modeled and so the D1 receptor stimulation. It was only the pertinent effect of dopamine/D1 receptor stimulation which was realized on the conductances of different kinds of receptors present in their computational models. In the study by Durstewitz et al. [25], a separate dopamine unit was atleast considered and shifts in the various cortical parameters were estimated based on the output activity of the dopamine unit. Yet, the mechanism of dopaminergic modulation was an oversimplification over the further detailed features associated with the building-up of dopamine content of DLPFC as well as the downstream activation of D1 receptors based on their sensitivity. However, in the present model, the presence of dopamine has been explicitly modeled in the form of dopamine content in the DLPFC, along with the subsequent stimulation of D1 receptors to observe their role in shaping the cortical activity. In addition, the dopamine content has been conceptualized here through a dynamical variable which introduces a more realistic temporal outlook to the level of dopamine as well as D1 receptor stimulation. In fact, this includes its coupling to other essential dynamics of cortical activities and midbrain stimulation inscribed in the present model design. Further, owing to its dynamical nature, noisy fluctuations in dopamine content, and thus in D1 receptor activity, are also being captured while they influence the resultant cortical activity. However, the earlier studies lack this facility due to the absence of an explicit mesocortical dynamics in their models framework.

The comparative differences in the framework of investigations have further repercussions on the description of robustness. The energy landscape description in the studies by Loh, Rolls and Deco [27, 63] relies on the differential responses of the attractor cortical network to varying D1 receptor stimulation. Since the global energy landscape in the present study is procured from the mesocortical circuit-level dynamics, the description of robustness not only relies on the cortical responses but also includes effects mediated through the involvement of mesocortical responses. Moreover, in the studies by Durestewitz et al. [61], the level of robustness was measured on the basis of the intensity of distractor stimulus needed to disrupt the existing sustained firing in the network. Although this strategy to measure robustness bears fair relationship with description of the inherent network’s robustness during working memory, this still stands as an indirect measure. On the other hand, the approach adopted here using global potential landscape draws a direct and detailed picture of the inherent robustness. An important point to note here is that this picture, in turn, establishes the underlying causality behind the findings made in the earlier studies and explains the observed rise in the intensity of distractor stimulus needed to disrupt the working memory state with rise in D1 receptor activation. In light of the above facts, the present model of stochastic mesocortical circuit dynamics bears a strong significance for dealing with a more comprehensive machinery underlying the working memory function.

### Fine-tuning of circuit robustness by excitatory-inhibitory activities

As mentioned above, the dependence of the circuit’s robustness on the level of D1 receptor activity observed is, in fact, the enhancement of the higher stable state or the sustained firing state of the system in comparison to its corresponding lower stable state or basal activity state with the increase in D1 receptor activity. However, when a comparative study is performed of the robustness of higher stable states among themselves in zones II (near Q), III and IV, an additional feature is observed in which there occurs an initial rise in the robustness of the state followed by a gradual decrease (inverse of the potential profile shown in Fig 6). This feature emerges mainly from the variation in the level of equilibrium excitatory activity in the higher stable states as the dopamine releasability varies from Q to R (See the solid upper line in the bifurcation plot Fig 3A). Evidently, the profile of variation in the robustness shares similarity with that of the equilibrium pyramidal activity in the higher stable state. This observation is in conformity with the established facts [27, 32, 63] where the level of excitatory pyramidal activity is critically involved in defining the depth of potential well belonging to a basin of attraction. Any factor which may lead to a decrease in their activity will lead to a decrease in the well depth resulting in increased stability of the basin of attraction [27, 32, 63]. In the present dynamical framework, the higher stable state is the bottom of a basin of attraction and, hence, its robustness is expected to be affected by the level of excitatory activity.

Nonetheless, one also witnesses here the presence of an unexpected behavior which raises a doubt regarding robustness to be solely governed by the excitatory activity. The point of maximum robustness or minimum potential of the higher stable state does not coincide with the point of maximum equilibrium pyramidal activity at point Q. The most robust configuration of the system at higher stable state lies at a point shifted away from the point Q towards a higher value of dopamine releasability. Certainly, it is an interesting finding as it contradicts what one would expect merely from the deterministic analysis of the system where the point Q of maximum intensity of sustained firing should be the point of maximum robustness. Within the dynamical framework, this leads to the question that if the variable *x*_1_ (excitatory neuron activity) is not the only one involved in shaping the robustness profile, what other variables are possibly behind this phenomenon?

To answer this question, we compare the bifurcation plots of the various variables. It is clear that the variable *x*_3_ (dopaminergic neuron activity) is following an identical profile to that of *x*_1_ in the higher stable state and its role is possibly redundant. The role of variable *d* (D1 receptor activity) has already been established in governing the overall global robustness of the circuit dynamics. Therefore, it seems that besides the variable *x*_1_, the variable *x*_2_ (inhibitory neuron activity) also contributes to the robustness. Indeed, it is noticeable that the point of minimum $U^{h}\left(\vec{x}\right)$ is in-between the maxima of *x*_1_ and *x*_2_ in zone III. This observation indicates that the point of maximum robustness is somewhat attracted towards the point of maximum equilibrium inhibitory activity, away from the point of maximum equilibrium excitatory activity, such that it lies somewhere at the tradeoff of the two activities. This leads to a possibility that there exists a subtle ratio of the equilibrium excitatory and inhibitory activities, which defines the optimal robustness of the sustained firing or working memory state. Since the D1 receptor activity coarsely governs the global robustness of the circuit dynamics, the involvement of such an excitatory-inhibitory ratio is being considered here as the fine tuning of the robustness. Nonetheless, it becomes necessary to further confirm this prediction through computational and experimental studies.

### Signal-to-noise ratio as a qualification for efficient working memory

Our results demonstrate that the sustained-firing states at lower dopamine release are not only associated with very low signals but also carry huge amount of noisy fluctuations (Fig 7). In fact, the relative magnitude of noise is so much so that the signal counterpart does not bear any substantial significance and, hence, the sustained-firing states do not qualify to be considered as an efficient working memory state. The same is witnessed for very high dopamine releasability where the signal gradually decreases and the relative noise content increases. Similar observations were made by Loh, Rolls and Deco [62, 63] where they used integrate-and-fire model along with varying the NMDA as well as GABA receptor conductances and investigated the signal-to-noise ratio in network firings. They suggest that low D1 receptor activity may lead to decrease in signal-to-noise ratio associated with sustained-firings in the attractor neural network and the quality of working memory.

Nonetheless, the present noisy framework of the model also gives rise to the possibility of describing the signal-to-noise ratio at the whole mesocortical circuit level. Since the circuit consists of the two principal functioning modules: DLPFC and Midbrain connected reciprocally, therefore, a parallel description of “local” signal-to-noise ratio in the two components during working memory activity collectively provides a “global” picture of the signal-to-noise ratio prevailing at the whole circuit level. In this context, our results (Fig 7) demonstrate that, in the optimal region of dopamine releasability, the signal-to-noise ratio together in PFC and midbrain are maximal, further indicating that the signal-to-noise ratio in the whole circuit is optimal. However, at abnormal levels of dopamine releasability, this ratio over the whole circuit gets poor. It may be noted that the description of signal-to-noise ratio at the circuit level is a completely seminal outcome of the present model framework and seems to possess potential strength in elucidating the quality of working memory at a more comprehensive level.

In the context of only signal, the inverted U-shaped profile of signal component obtained in our study through the deterministic analysis is similar to that observed in the study of object working memory performed by Brunel and Wang [23]. They employed an attractor neural network based on integrate-and-fire neurons and the dopaminergic modulation of the network activity was realized by making the NMDA receptor conductance dependent on D1 receptor activity. The establishment of working memory-associated sustained firing activity due to strong recurrent inhibition in the cortical network shown in their work is very similar to the description provided in the present study using the interplay of feed-forward activation and feed-forward inhibition. However, an important point to note is that the signal profile in their exploration was obtained through varying the D1 receptor activity independently. On the contrary, in the present framework, the cortical activity itself is reciprocally involved in affecting the dopamine release from midbrain and the subsequent change in D1 receptor activity. Therefore, the notable differences in the signal profiles obtained from the two studies (compare Fig 3A with Fig 10 of [23]) owe to the inclusion of the mesocortical dynamics in the present study.

### Clinical implications and predictions: Ageing, acute stress and schizophrenia

Be it ageing, stress or neuropsychiatric disorders, such as schizophrenia, dysfunctions in dopaminergic system and alterations in D1 receptor activity are pivotal to working memory impairment. Therefore, we consider here these clinical conditions which are associated with strikingly different dopamine levels and D1 receptor stimulation in the DLPFC.

**Ageing: A case of hypodopaminergic DLPFC with suboptimal D1 receptor stimulation**. In the study performed by Bartus et al. [11] on aged rhesus monkeys, a poor working memory performance during delayed-response task was observed, in the sense that their working memory was vulnerable to disruption by distractors provided in the delay-interval. A similar situation appears if lesions are caused to the DLPFC of rather young monkeys [64, 65]. Employing a spatial delayed-response task with a two-well scheme, Rapp and Amaral [66] showed that the aged monkeys could not retain working memory even for a five second delay interval during the task and often collapsed pre-maturely. In a recent fMRI study in aged human subjects [67], reduced top-down suppression in sensory interference has been shown to be involved in impaired working memory. Similarly, altered working memory in elderly age human candidates has also been confirmed on the basis of low priming observed in immediate- and delayed-recall tests [68].

The neurobiological basis of ageing has been extensively associated with the declined neuromodulation of cortical activity by catecholamines, such as dopamine. Brozoski et al. [69] using 6-hydroxydopamine lesion to the monkey DLPFC showed that dopamine depletion heavily impairs the spatial working memory. A naturally-occurring depletion in dopamine has been demonstrated to impact working memory performance in aged monkeys [11] and rats [70]. Degeneration of dopamine nuclei causing dopamine depletion has also been reported in aged monkeys [71, 72]. Ameliorating effects of low doses of D1 receptor full agonists A77636 or SKF81297 on the poor spatial working memory in aged monkeys has been reported in the study by Cai and Arnsten [73]. However, the improvement in cognitive ability was reversed by high doses of D1 receptor agonists. Study involving selective dopamine antagonists has also suggested low D1 receptor activation in age-related working memory deficit [9]. Loss of dopamine receptors and alteration in the dopamine-binding sites in the receptor in ageing has been confirmed in post mortem studies [74] and PET studies [75] in humans.

The present study effectively explains the poor performance on the basis of the dynamics of mesocortical circuit and its robustness. It suggests that the dopamine releasability from the excited dopaminergic neurons might go low which may eventually lead to a low level of D1 receptor activity in the DLPFC. Such a situation can be realized in the present framework in zone II (near P) where the dopamine releasability parameter *W*_34_ and the corresponding equilibrium D1 receptor activity are significantly low (Fig 3D). Under such conditions, it is observed that the lower stable state (basal-activity state) of the system is more robust in comparison to the corresponding higher stable state (working memory state). Also, the higher stable state has a very shallow potential well with a significantly small potential barrier to the lower stable state. Here, as the cue input causes a transition of the system from lower to higher stable state where working memory is formed, the system in the higher stable state is highly vulnerable to jump back to the lower stable state in the presence of noisy perturbations in the circuit. As a consequence, during the delay period, the established working memory will not be able to sustain itself for a sufficient time interval and the memory soon gets destroyed by the consistent background noise. This is also reflected by the extremely low signal-to-noise ratio associated with the working memory state under such conditions (Fig 7). Moreover, the poor signal content signifying low neural activity in the DLPFC during delay interval is an added debilitating factor resulting in cognitive deficit.

As evident, the findings provide a straightforward explanation to the experimentally-observed vulnerability of working memory state towards disruption and elucidate the role of D1 receptor stimulation in ageing. The dose-dependent effect of D1 receptor full agonists observed in the study by Cai and Arnsten [73] can be clearly explained on the basis of signal-to-noise ratio of working memory (Fig 7) as well as the associated potential energy landscape (Fig 5). As the D1 receptor stimulation increases (Fig 3D), the signal-to-noise ratio increases with deepening of the sustained-firing associated basin of attraction. This leads to improvement of the working memory at low doses of D1 receptor agonist. However after reaching an optimum level, the signal-to-noise ratio starts decreasing with a gradual increase in the shallowness of the sustained-firing associated basin of attraction, which explains the observed adverse effect of high doses of D1 receptor agonist on the cognitive ability.

In a recent computational study by Rolls and Deco [32] using cortical attractor network, it was shown that sustained-firing activity owing to its shallow basin of attraction is vulnerable to collapse to the spontaneous state under low D1 receptor stimulation. Decreased firing rate and low signal-to-noise ratio have also been shown associated with impaired working memory in ageing. Nonetheless, the present findings elucidate the underlying mechanism of age-related cognitive deficit at a more comprehensive level of functional machinery viz. mesocortical dynamics involving an explicit role of dopamine and D1 receptor stimulation as compared to the previous study [32].

**Acute stress: A case of hyperdopaminergic DLPFC with supraoptimal D1 receptor stimulation**. In the study by Murphy et al. [22] using anxiogenic beta-carboline FG7142 to achieve mild stress-related biochemical effects in rats and monkeys, deterioration of working memory activity was demonstrated under high dopamine turnover in PFC. Moreover, these cognitive impairments were alleviated by use of the dopamine receptor antagonist SCH23390, haloperidol and clozapine or the drug RO15-1788 causing suppression of dopamine turnover. Working memory impairment was also observed by Arnsten and Goldman-Rakic [76] when monkeys acutely exposed to loud noise of 100-110 dB intensity performed poorly in spatial delayed-response task. Further treatment with D1 receptor antagonist SCH 23390 or drugs leading to suppression of dopamine turnover in DLPFC, such as clonidine, were shown to improve the stress-induced cognitive impairment. The adverse effect of acute stress on working memory in humans has been studied by Qin et al. [12] where healthy subjects exposed to acute psychological stress underwent numerical N-back task and their neural activity was recorded using block-design fMRI. The results revealed hypofrontality in the DLPFC and was attributed to supraoptimal activation of D1 receptors under high dopamine content of DLPFC.

In the present study, such a situation is implied by the high level of dopamine releasability *W*_34_, where high D1 receptor activity is obtained in the farther region of zone IV (Fig 3D). Here, the potential well of the lower stable state becomes extremely shallow in comparison to the corresponding higher stable state (Fig 5). Even noise can cause a transition of the system to the sustained-firing state (higher stable state) when no cue input has been provided. This demonstrates a spontaneous formation of working memory, where no call for its genuine formation has been made by any desired external input. Coincidently, the higher stable state is also not enough robust in itself (however relatively more robust than the corresponding lower stable state) and a sufficiently enough noise can cause the transition of the system to the basal-activity state. Even if a working memory is formed in response to a desired external cue, it is not able to sustain itself during the delay. This can also be observed through low signal-to-noise ratio associated with the sustained-firing state (Fig 7).

Overall, what one may observe here is the constant transitions of the system from memory-less to memory-loaded state. In other words, task-irrelevant or goal-irrelevant memory will be often formed and soon dissolved, along with a weak capacity to hold any relevant memory formed for a sufficient time interval. These observations together posit a possible mechanism underlying the disruption of working memory during delay interval in the DLPFC of rats and monkeys under mild stress, as reported in the above-mentioned experimental studies [22, 76]. It also explains the alleviating effects of dopamine receptor antagonist as it causes leftward shift of the system to optimal region-associated lower D1 receptor stimulation (Fig 3D) and subsequent increase in signal-to-noise ratio along with the deepening of basins of attractions for spontaneous as well as sustained firing states. Moreover, the acute stress-induced hypofrontality in DLPFC recorded in the study by Qin et al [12] is also explained by the low signal observed in the region of high dopamine releasability (Fig 3A).

Nonetheless, it is important to note that the seemingly similar working memory impairments in old-age and acute stress conditions emanate from substantially different mechanistic routes. In the case of old age-related conditions, weakly robust sustained-firing state highly sensitive to noise/distractions is the primary reason behind the impaired working memory. On the contrary, in the case of acute stress conditions, not only the sensitivity of sustained-firing state to albeit strong noise/distractions but also the unpredictable spontaneous induction of task-irrelevant sustained-firing patterns during delay interval together lay causal grounds for the cognitive deficit.

**Schizophrenia: A case of hypodopaminergic DLPFC with upregulated D1 receptor density**. Cognitive impairment involving poor ability of retention and manipulation of working memory-associated representations during delay interval is one of the most remarkable symptoms of schizophrenia. In the study by Park and Holzman [77], schizophrenic patients were found to exhibit highly vulnerable working memory and high susceptibility to perseverative errors during spatial occulomotor delayed-response task. Similar observations were made in the study by Gold et al. [78] demonstrating poor performance by schizophrenic patients during Wisconsin Card Sorting Task. Neuroimaging studies have also shown abnormal activation (hypofrontality) of DLPFC in patients with schizophrenia during working memory tasks, such as N-back task [79], suggesting pathological implications in cognitive deficit in schizophrenia.

A large body of literature suggests the cortical dopamine dysfunction as a primary reason behind cognitive deficits in schizophrenic patients. Several indirect evidences, such as reduced dopaminergic transmission during cognitive tasks in schizophrenic patients [53] and presence of low homovanillic acid in cerebrospinal fluid [80], indicate declined presynaptic dopamine activity in DLPFC. Postmortem studies [54] have provided a more direct evidence of dopamine depletion in DLPFC due to reduced cortical innervations by dopaminergic afferents in patients with schizophrenia. At least, these evidences have confirmed to a great extent that hypodopaminergic condition indeed prevails in the DLPFC in the patients with schizophrenia. However, it has not been yet confirmed whether low or high D1 receptor stimulation is involved in cognitive deficit. In this regard, in-vivo investigations of density of D1 receptors in DLPFC of drug-naïve schizophrenic patients performed by Abi-Dargham and colleagues [81, 82] using selectively-binding [^11^C]NNC 112 radiotracers has provided an extremely strong and direct evidence of elevated D1 receptor density in the cortical region, especially the DLPFC. As a result, this finding has lead to the postulation of three possible kinds of models [81–83] to elucidate the role of altered D1 receptor activation in working memory impairment in schizophrenia.

According to the first model, rise in the density of D1 receptors is merely a secondary response to chronic deficiency of dopamine and occurs as an adaptive measure, although ineffective, to compensate for the decline in D1 receptor stimulation. Therefore, this model suggests that working memory impairment in schizophrenia is associated with low D1 receptor stimulation and use of dopamine agonists or atypical drugs leading to enhanced prefrontal dopamine release may alleviate the impairment. This interpretation has been indirectly supported by many experimental evidences and has been a subject of extensive investigation in many earlier computational studies [27, 31, 62, 63, 84]. The present study captures this situation in zone II (see bifurcation plot (Fig 3D)) which belongs to low dopamine releasability (hypodopaminergic DLPFC) and low D1 receptor stimulation. In this region, a too shallow potential well associated with sustained firing (see Fig 5), hypofrontality (see Fig 3A) and low signal-to-noise ratio (see Fig 7) together hamper the working memory state. Nonetheless, this interpretation also suggests implication of ageing conditions into schizophrenia. However, there lies a remarkable difference between the two clinical conditions, where ageing is associated with a marked decrease in D1 receptor density (downregulation) [74, 75].

The second model suggests that rise in the D1 receptor density may possibly be the primary reason behind the cognitive deficit in schizophrenia. Release of dopamine in DLPFC under the demands of cognitive task or stress condition may lead to the supraoptimal stimulation of D1 receptors, which may subsequently engender working memory impairments. This interpretation is corroborated by the working memory impairments observed under the conditions of high dopamine release or presence of dopamine agonists in animal models which involve high D1 receptor stimulation [69, 73]. However, this interpretation is still a subject of investigation and has not gained enough clinical support until now.

The third model is an amalgam of the above two models. It states that rise in the D1 receptor density is indeed a compensatory mechanism to chronic dopamine depletion. However, the increased density and sensitivity of cortical D1 receptors may make it easier to attain hyperstimulation when cortical dopamine release is elevated under cognitive tasks or stress conditions. This may lead to the working memory impairment observed in patients with schizophrenia. The ameliorating effects of atypical antipsychotics on schizophrenia-associated cognitive deficits provide a strong support to this interpretation (see [82]) and bears potential clinical implications.

Our study captures the condition of high D1 receptor stimulation in zone IV of the bifurcation plot (Fig 3D). However, this region is further associated with high dopamine releasability depicting hyperdopaminergic state of DLPFC under the present model framework. Here, our argument is that high D1 receptor stimulation observed either through normal D1 receptor density under high dopamine releasability or due to the upregulated cortical D1 receptor density would make a similar impact on the cortical activity. It is the resultant D1 receptor activity which primarily affects the activity of pyramidal neurons and inhibitory interneurons in the cortical network at the cellular/ physiological level. This argument may stand valid as long as D1 receptor stimulation is considered as the only means of dopaminergic modulation. Moreover, this argument also needs that there are no other damaging effects on the cyotarchitecture of the DLPFC inflicted by the two routes leading to supraoptimal D1 receptor stimulation. Both these conditions for the argument to remain valid are found to be satisfied atleast in the present model framework. Therefore, it would not be inappropriate to say that the mechanisms for cognitive deficits that have been elucidated here for high D1 receptor stimulation associated with high dopamine releasability (hyperdopaminergic DLPFC) may also illustrate the mechanisms underlying the cognitive deficits observed under schizophrenia. Accordingly, spontaneous formation of task-irrelevant working memory and weakly robust noise-sensitive task-relevant working memory associated with this region together provides a plausible mechanism for the cognitive deficits observed under the interpretation of the third model.

Further, since zone IV in our study has been attributed to acute stress condition, this stems out various possible implications of acute stress to schizophrenia which already has been a subject of immense discussion in the past literature. Working memory impairments under acute stress is found to be associated with higher-order cognitive dysfunctions observed in various psychiatric disorders, including schizophrenia [12, 85]. Role of acute stress in exacerbating the psychiatric events and associated decline in cognitive ability in schizophrenic patients has also been highlighted [22, 86].

## Conclusion

The model of the closed loop mesocortical circuit [28], which elucidates the role of the midbrain region in the dopaminergic modulation of the DLPFC activity and working memory through D1 receptor stimulation is re-examined. The functioning of the prefronto-mesoprefrontal system is studied in the presence of background noise emanating from various natural sources which constantly affects the circuit dynamics. The global potential landscape of the circuit dynamics is constructed to gain insight into the steady state properties of the system. Also, signal-to-noise ratio in the cortical as well as midbrain activity is obtained based on the global potential landscape which describes the robustness of working memory during delay-period.

The energy landscape reveals that high D1 receptor stimulation favors the sustained-firing state over the basal-activity state of the system during the delay period. However, the circuit’s robustness is further fine-tuned in a nonlinear fashion by the level of excitatory and inhibitory activities underlying the sustained-firing state such that the robustness of sustained-firing state during delay period exhibits an inverted-U shaped profile with respect to the level of D1 receptor stimulation. Therefore, the optimal region of robustness of working memory during delay period is obtained only in a certain intermediate range of D1 receptor stimulation. Too low or too high D1 receptor stimulation may lead to poor robustness of working memory. This is also in conformity with the inverted-U shaped profile of signal-to-noise ratio associated with the working memory state. Moreover, the study predicts that the most robust working memory is formed possibly at a subtle ratio of the excitatory and inhibitory activity in the sustained firing state achieved at a critical level of D1 receptor stimulation.

Based on the knowledge of the circuit’s robustness profile, the study further suggests possible mechanistic routes to the cognitive deficits observed in ageing, acute stress and schizophrenia. Under ageing- associated hypodopaminergic condition of DLPFC and consequent suboptimal D1 receptor stimulation, it is shown that the weakly robust sustained-firing state highly vulnerable to disruption in the presence of background noise is the primary reason behind the observed impaired working memory. However, under acute stress-associated hyperdopaminergic condition of DLPFC with subsequent supraoptimal D1 receptor stimulation, the findings suggest that vulnerability of sustained-firing state to albeit strong noise/distractions as well as the unpredictable spontaneous formation of task-irrelevant sustained-firing patterns during delay interval may potentially lead to the cognitive deficit. In the case of schizophrenia which is associated with hypodopaminergic DLPFC and concomitant upregulation of cortical D1 receptors, three plausible mechanisms of cognitive deficits [81–83] relying on either suboptimal or supraoptimal D1 receptor stimulations are discussed in the context of the present study. Consequently, the present study suggests that the mechanisms which have been shown to be involved in cognitive deficits under age-related and acute stress conditions may also have possible implications for the cognitive deficits observed in patients with schizophrenia.

As compared to the earlier computational studies [25–27, 61, 62] regarding robustness of working memory, the present study deals with a more comprehensive functional machinery underlying the working memory function. The presence of dopamine content in the DLPFC and subsequent D1 receptor stimulation are explicitly modeled and are conceptualized in dynamical forms coupled to cortical and midbrain activities. Accordingly, even the noisy fluctuations in dopamine and D1 receptor stimulation are also accounted for in the present framework. Therefore, the description of robustness of working memory based on the global potential landscape in the present study not only involves factors associated with cortical dynamics but also the mesocortical dynamics. In light of these facts, the present model of stochastic mesocortical dynamics undoubtedly marks its potential utility over other earlier studies to elucidate a more close-to-reality description of the dopamine-influenced driving forces involved in shaping the various attributes of the cortical activity associated with working memory. Nonetheless, many possible advances in the model framework capturing a relatively larger functioning apparatus underlying working memory awaits, where nigrostriatal pathways may also be considered to take into account the role of cortical as well as striatal dynamics along with the dopaminergic midbrain activity. Also, role of other neurotransmitters, such as epinephrine and serotonin, may also be incorporated in the future design of the model to acquire a more integrated understanding of the working memory function and its deficit in various clinical conditions.
