# Correction: Stochastic Mesocortical Dynamics and Robustness of Working Memory during Delay-Period

**DOI:** 10.1371/journal.pone.0205615

**Published:** 2018-10-04

**Authors:** Melissa Reneaux, Rahul Gupta, Karmeshu

The following information is missing from the Funding section: The authors received funding from the Department of Science and Technology, India, under the DST PURSE-II grant towards publication charges.
